# 3-Amino-1-(thio­phen-2-yl)-9,10-dihydro­phenanthrene-2,4-dicarbonitrile

**DOI:** 10.1107/S1600536812010033

**Published:** 2012-03-10

**Authors:** Abdulrahman O. Al-Youbi, Abdullah M. Asiri, Hassan M. Faidallah, Seik Weng Ng, Edward R. T. Tiekink

**Affiliations:** a Chemistry Department, Faculty of Science, King Abdulaziz University, PO Box 80203, Jeddah, Saudi Arabia; b The Center of Excellence for Advanced Materials Research, King Abdulaziz University, Jeddah, PO Box 80203, Saudi Arabia; c Department of Chemistry, University of Malaya, 50603 Kuala Lumpur, Malaysia

## Abstract

In the title compound, C_20_H_13_N_3_S, the partially saturated ring adopts a twisted half-boat conformation with the methyl­ene C atom closest to the amino­benzene ring lying 0.690 (6) Å out of the plane defined by the five remaining atoms. The dihydro­phenanthrene residue has a folded conformation [dihedral angle between the outer benzene rings = 26.27 (18)°]. The thio­phen-2-yl ring forms a dihedral angle of 63.76 (19)° with the benzene ring to which it is attached. In the crystal, inversion dimers linked by pairs of N—H⋯N hydrogen bonds generate *R*_2_^2^(12) loops. The dimers are linked into layers in the *bc* plane by weak C—H⋯π inter­actions. The thio­phen-2-yl ring is disordered over two essentially coplanar but opposite orientations in a 0.918 (4):0.082 (4) ratio.

## Related literature

For background to the biological activity of related dicarbonitrile compounds, see: Aly *et al.* (1991 ▶); Rostom *et al.* (2011 ▶). For related structures, see: Asiri *et al.* (2011*a* ▶,*b* ▶).
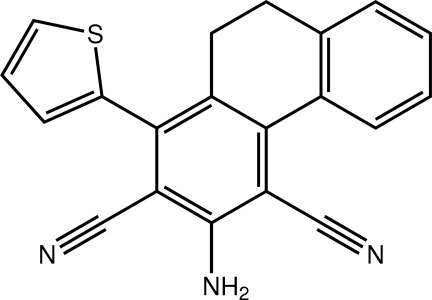


## Experimental

### 

#### Crystal data

C_20_H_13_N_3_S
*M* *_r_* = 327.39
Monoclinic, 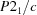

*a* = 9.7882 (10) Å
*b* = 7.1199 (7) Å
*c* = 22.746 (3) Å
β = 93.171 (11)°
*V* = 1582.8 (3) Å^3^
*Z* = 4
Mo *K*α radiation
μ = 0.21 mm^−1^
*T* = 100 K
0.30 × 0.06 × 0.03 mm

#### Data collection

Agilent SuperNova Dual diffractometer with an Atlas detector
Absorption correction: multi-scan (*CrysAlis PRO*; Agilent, 2011 ▶) *T*_min_ = 0.940, *T*_max_ = 0.994
5527 measured reflections
2805 independent reflections
1778 reflections with *I* > 2σ(*I*)
*R*_int_ = 0.056

#### Refinement

*R*[*F* ^2^ > 2σ(*F* ^2^)] = 0.066
*wR*(*F* ^2^) = 0.164
*S* = 1.03
2805 reflections
236 parameters
56 restraints
H atoms treated by a mixture of independent and constrained refinement
Δρ_max_ = 0.67 e Å^−3^
Δρ_min_ = −0.55 e Å^−3^

### 

Data collection: *CrysAlis PRO* (Agilent, 2011 ▶); cell refinement: *CrysAlis PRO*; data reduction: *CrysAlis PRO*; program(s) used to solve structure: *SHELXS97* (Sheldrick, 2008 ▶); program(s) used to refine structure: *SHELXL97* (Sheldrick, 2008 ▶); molecular graphics: *ORTEP-3* (Farrugia, 1997 ▶) and *DIAMOND* (Brandenburg, 2006 ▶); software used to prepare material for publication: *publCIF* (Westrip, 2010 ▶).

## Supplementary Material

Crystal structure: contains datablock(s) global, I. DOI: 10.1107/S1600536812010033/hb6670sup1.cif

Structure factors: contains datablock(s) I. DOI: 10.1107/S1600536812010033/hb6670Isup2.hkl

Supplementary material file. DOI: 10.1107/S1600536812010033/hb6670Isup3.cml

Additional supplementary materials:  crystallographic information; 3D view; checkCIF report

## Figures and Tables

**Table 1:** Hydrogen-bond geometry (Å, °) *Cg*1 is the centroid of the C4–C9 ring.

*D*—H⋯*A*	*D*—H	H⋯*A*	*D*⋯*A*	*D*—H⋯*A*
N2—H1⋯N3^i^	0.88 (3)	2.18 (3)	3.016 (5)	160 (3)
C6—H6⋯*Cg*1^ii^	0.95	2.85	3.660 (5)	144

Symmetry codes: (i) 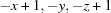; (ii) 
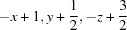
.

**Fig. 1.**
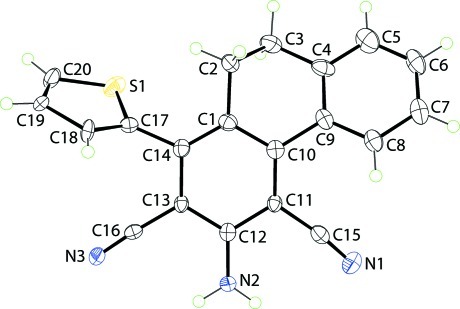
The molecular structure of (I) showing displacement ellipsoids at the 50% probability level.

**Fig. 2.**
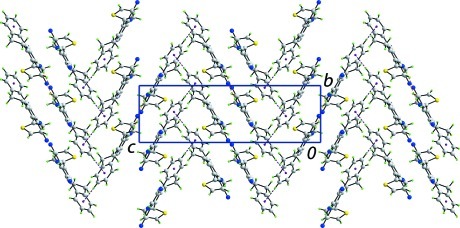
A view of the supramolecular layer in the bc plane in (I). The C—H···O and C—H···π interactions are shown as orange and purple dashed lines, respectively.

**Fig. 3.**
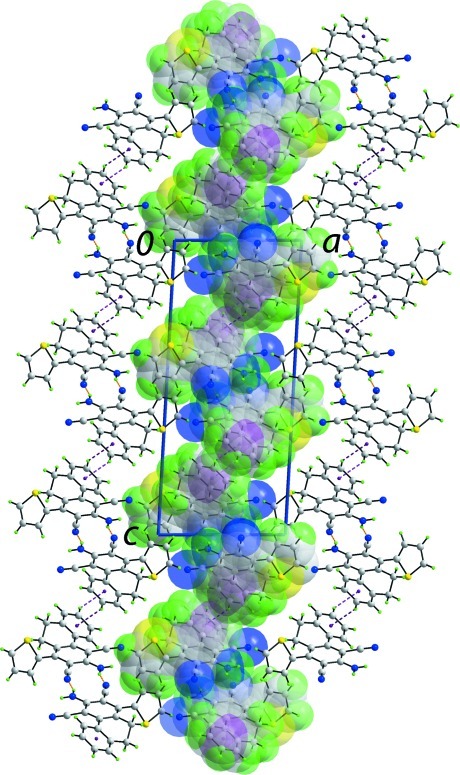
A view in projection down the b axis of the unit-cell contents of (I) showing the stacking of layers. The C—H···O and C—H···π interactions are shown as orange and purple dashed lines, respectively. One layer is highlighted in space-filling mode.

## supplementary crystallographic information

### Comment

The crystallographic investigation of the title compound, (I), was motivated by reports of the biological activity of related compounds (Aly *et al.*, 1991; Rostom *et al.*, 2011) and allied crystal structure investigations (Asiri *et al.*, 2011*a*; Asiri *et al.*, 2011*b*).

In (I), Fig. 1, the partially saturated ring adopts a twisted half boat conformation with the C2 atom lying 0.690 (6) Å out of the plane defined by the five remaining atoms [r.m.s. deviation = 0.1032 Å; maximum deviations = 0.076 (3) Å for the C9 atom and -0.160 (3) Å for the C10 atom]. The dihedral angle between the adjacent benzene rings = 26.27 (18)° indicating a fold in the molecule. The thiophen-2-yl ring forms a dihedral angle of 63.76 (19)° with the benzene to which it is attached.

In the crystal packing, centrosymmetric aggregates are formed *via* N—H···N hydrogen bonds leading to 12-membered {···HNC_3_N}_2_ synthons, Table 1. These are linked into layers in the *bc* plane by C—H···π interactions, Fig. 2 and Table 1. These stack along the *a* axis with no specific interactions between them.

### Experimental

A mixture of thiophene-2-cabaldehyde (1.1 g, 10 mmol), 1-tetralone (1.46 g, 10 mmol), malononitrile (0.66 g, 10 mmol) and ammonium acetate (6.2 g, 80 mmol) in absolute ethanol (50 ml) was refluxed for 6 h. The reaction mixture was allowed to cool, and the formed precipitate was filtered, washed with water, dried and recrystallized from ethanol as orange prisms. Yield: 69%. *M*.pt: 451–453 K.

### Refinement

Carbon-bound H-atoms were placed in calculated positions [C—H = 0.95 to 0.99 Å, *U*_iso_(H) = 1.2*U*_eq_(C)] and were included in the refinement in the riding model approximation.

The amino H-atoms were located in a difference Fourier map, and were refined with a distance restraint of N—H = 0.88±0.01 Å; *U*_iso_ were refined.

The thienyl ring is disordered over two positions in a 0.918 (4): 0.082 (4) ratio. The S—C distances were restrained to 1.71±0.01 Å, the formal C—C single-bond distances to 1.42±0.01 Å and the formal C═C double-bond distances to 1.42±0.01 Å. Additionally, the 1,3-related distances were restrained to within 0.01 Å of each other. Because pairs of atoms are close to each other, the *U*_aniso_ of the C18' atom were equated to those of the S1 atom (as well as the C19'/C20, C20'/C19' and C18'/S1 pairs). The anisotropic displacement parameters were tightly restrained to be nearly isotropic.

### Crystal data

**Table:**

C_20_H_13_N_3_S	*F*(000) = 680
*M**_r_* = 327.39	*D*_x_ = 1.374 Mg m^−^^3^
Monoclinic, *P*2_1_/*c*	Mo *K*α radiation, λ = 0.71073 Å
Hall symbol: -P 2ybc	Cell parameters from 1132 reflections
*a* = 9.7882 (10) Å	θ = 2.7–25.0°
*b* = 7.1199 (7) Å	µ = 0.21 mm^−^^1^
*c* = 22.746 (3) Å	*T* = 100 K
β = 93.171 (11)°	Prism, orange
*V* = 1582.8 (3) Å^3^	0.30 × 0.06 × 0.03 mm
*Z* = 4	

### Data collection

**Table:**

Agilent SuperNova Dual diffractometer with an Atlas detector	2805 independent reflections
Radiation source: SuperNova (Mo) X-ray Source	1778 reflections with *I* > 2σ(*I*)
Mirror monochromator	*R*_int_ = 0.056
Detector resolution: 10.4041 pixels mm^-1^	θ_max_ = 25.1°, θ_min_ = 2.7°
ω scan	*h* = −9→11
Absorption correction: multi-scan (*CrysAlis PRO*; Agilent, 2011)	*k* = −8→6
*T*_min_ = 0.940, *T*_max_ = 0.994	*l* = −19→27
5527 measured reflections	

### Refinement

**Table:**

Refinement on *F*^2^	Primary atom site location: structure-invariant direct methods
Least-squares matrix: full	Secondary atom site location: difference Fourier map
*R*[*F*^2^ > 2σ(*F*^2^)] = 0.066	Hydrogen site location: inferred from neighbouring sites
*wR*(*F*^2^) = 0.164	H atoms treated by a mixture of independent and constrained refinement
*S* = 1.03	*w* = 1/[σ^2^(*F*_o_^2^) + (0.0559*P*)^2^ + 1.2744*P*] where *P* = (*F*_o_^2^ + 2*F*_c_^2^)/3
2805 reflections	(Δ/σ)_max_ = 0.001
236 parameters	Δρ_max_ = 0.67 e Å^−^^3^
56 restraints	Δρ_min_ = −0.55 e Å^−^^3^

### Fractional atomic coordinates and isotropic or equivalent isotropic displacement parameters (Å^2^)

**Table:**

	*x*	*y*	*z*	*U*_iso_*/*U*_eq_	Occ. (<1)
S1	0.98458 (11)	0.25812 (17)	0.64193 (6)	0.0331 (4)	0.918 (4)
S1'	0.976 (2)	0.275 (3)	0.5229 (7)	0.0358 (13)	0.082 (4)
N1	0.2456 (3)	0.6816 (5)	0.61810 (16)	0.0329 (9)	
N2	0.3958 (3)	0.3401 (5)	0.54813 (14)	0.0213 (7)	
H1	0.402 (4)	0.236 (3)	0.5276 (15)	0.026*	
H2	0.3108 (16)	0.373 (5)	0.5535 (17)	0.026*	
N3	0.6533 (3)	0.0358 (5)	0.51041 (15)	0.0255 (8)	
C1	0.7477 (4)	0.6177 (5)	0.61652 (16)	0.0213 (9)	
C2	0.8711 (4)	0.7249 (5)	0.64154 (18)	0.0262 (9)	
H2A	0.8904	0.8322	0.6156	0.031*	
H2B	0.9523	0.6418	0.6438	0.031*	
C3	0.8422 (4)	0.7966 (6)	0.70321 (18)	0.0329 (11)	
H3A	0.8278	0.6888	0.7297	0.039*	
H3B	0.9215	0.8696	0.7196	0.039*	
C4	0.7144 (4)	0.9209 (5)	0.69949 (19)	0.0295 (10)	
C5	0.7052 (5)	1.0784 (6)	0.73318 (19)	0.0380 (11)	
H5	0.7768	1.1082	0.7615	0.046*	
C6	0.5919 (4)	1.1956 (6)	0.72634 (19)	0.0329 (11)	
H6	0.5848	1.3024	0.7510	0.040*	
C7	0.4905 (4)	1.1578 (5)	0.68423 (18)	0.0289 (10)	
H7	0.4145	1.2402	0.6792	0.035*	
C8	0.4980 (4)	0.9990 (5)	0.64865 (18)	0.0260 (10)	
H8	0.4288	0.9752	0.6187	0.031*	
C9	0.6089 (4)	0.8743 (5)	0.65742 (16)	0.0221 (9)	
C10	0.6174 (4)	0.6957 (5)	0.62403 (16)	0.0201 (9)	
C11	0.5006 (3)	0.5995 (5)	0.60216 (16)	0.0173 (8)	
C12	0.5094 (4)	0.4290 (5)	0.57009 (16)	0.0184 (8)	
C13	0.6410 (3)	0.3549 (5)	0.56377 (16)	0.0170 (8)	
C14	0.7610 (3)	0.4495 (5)	0.58825 (16)	0.0202 (9)	
C15	0.3602 (4)	0.6548 (5)	0.61278 (17)	0.0237 (9)	
C16	0.6518 (3)	0.1773 (5)	0.53443 (16)	0.0193 (9)	
C17	0.8889 (3)	0.3421 (4)	0.58171 (17)	0.0234 (9)	
C18	0.9449 (5)	0.2974 (7)	0.5317 (2)	0.0358 (13)	0.918 (4)
H18	0.9073	0.3340	0.4940	0.043*	0.918 (4)
C18'	0.953 (2)	0.282 (3)	0.6333 (6)	0.0331 (4)	0.08
H18'	0.9194	0.3055	0.6710	0.040*	0.082 (4)
C19	1.0686 (4)	0.1878 (6)	0.5402 (2)	0.0325 (13)	0.918 (4)
H19	1.1203	0.1430	0.5089	0.039*	0.918 (4)
C19'	1.0749 (19)	0.179 (3)	0.6240 (13)	0.0361 (13)	0.082 (4)
H19'	1.1321	0.1274	0.6549	0.043*	0.082 (4)
C20	1.1022 (4)	0.1564 (6)	0.5979 (2)	0.0361 (13)	0.918 (4)
H20	1.1802	0.0875	0.6122	0.043*	0.918 (4)
C20'	1.1006 (14)	0.1645 (15)	0.5660 (15)	0.0325 (13)	0.08
H20'	1.1771	0.1011	0.5514	0.039*	0.082 (4)

### Atomic displacement parameters (Å^2^)

**Table:**

	*U*^11^	*U*^22^	*U*^33^	*U*^12^	*U*^13^	*U*^23^
S1	0.0230 (7)	0.0336 (7)	0.0420 (8)	0.0037 (5)	−0.0030 (5)	0.0073 (6)
S1'	0.021 (3)	0.045 (3)	0.043 (3)	0.005 (2)	0.015 (2)	−0.020 (2)
N1	0.025 (2)	0.035 (2)	0.038 (2)	0.0062 (16)	0.0029 (17)	0.0003 (17)
N2	0.0165 (16)	0.0228 (17)	0.0244 (19)	−0.0001 (15)	−0.0003 (14)	−0.0056 (15)
N3	0.0169 (17)	0.0238 (19)	0.036 (2)	0.0003 (14)	0.0039 (15)	−0.0071 (17)
C1	0.022 (2)	0.021 (2)	0.021 (2)	−0.0035 (17)	0.0023 (16)	0.0002 (17)
C2	0.024 (2)	0.023 (2)	0.032 (2)	−0.0028 (18)	0.0044 (18)	−0.0083 (19)
C3	0.032 (2)	0.035 (2)	0.031 (3)	−0.005 (2)	−0.0030 (19)	−0.010 (2)
C4	0.034 (2)	0.021 (2)	0.032 (3)	−0.0097 (19)	−0.0055 (19)	0.0035 (19)
C5	0.047 (3)	0.041 (3)	0.026 (3)	−0.004 (2)	0.012 (2)	−0.006 (2)
C6	0.047 (3)	0.027 (2)	0.026 (2)	−0.002 (2)	0.018 (2)	−0.007 (2)
C7	0.040 (2)	0.020 (2)	0.029 (2)	0.0020 (19)	0.016 (2)	0.0036 (19)
C8	0.033 (2)	0.022 (2)	0.023 (2)	−0.0007 (19)	0.0085 (18)	−0.0005 (18)
C9	0.030 (2)	0.0173 (19)	0.020 (2)	−0.0026 (18)	0.0090 (17)	0.0006 (17)
C10	0.023 (2)	0.019 (2)	0.018 (2)	−0.0003 (17)	0.0038 (16)	0.0037 (16)
C11	0.0177 (19)	0.0141 (18)	0.021 (2)	0.0009 (16)	0.0038 (16)	0.0016 (16)
C12	0.021 (2)	0.0190 (19)	0.015 (2)	0.0010 (17)	0.0041 (16)	0.0029 (16)
C13	0.0161 (19)	0.0154 (18)	0.020 (2)	0.0000 (16)	0.0063 (15)	0.0004 (16)
C14	0.020 (2)	0.020 (2)	0.021 (2)	−0.0004 (16)	0.0051 (16)	0.0016 (17)
C15	0.032 (2)	0.018 (2)	0.020 (2)	−0.0007 (18)	−0.0020 (18)	−0.0004 (17)
C16	0.0144 (19)	0.022 (2)	0.021 (2)	−0.0030 (16)	0.0006 (16)	0.0018 (18)
C17	0.0176 (19)	0.023 (2)	0.030 (2)	−0.0044 (17)	0.0021 (17)	−0.0049 (18)
C18	0.021 (3)	0.045 (3)	0.043 (3)	0.005 (2)	0.015 (2)	−0.020 (2)
C18'	0.0230 (7)	0.0336 (7)	0.0420 (8)	0.0037 (5)	−0.0030 (5)	0.0073 (6)
C19	0.011 (2)	0.033 (2)	0.053 (3)	0.0018 (19)	0.000 (2)	−0.021 (2)
C19'	0.018 (2)	0.027 (2)	0.064 (4)	0.0064 (19)	0.004 (2)	−0.008 (3)
C20	0.018 (2)	0.027 (2)	0.064 (4)	0.0064 (19)	0.004 (2)	−0.008 (3)
C20'	0.011 (2)	0.033 (2)	0.053 (3)	0.0018 (19)	0.000 (2)	−0.021 (2)

### Geometric parameters (Å, º)

**Table:**

S1—C17	1.723 (4)	C7—C8	1.395 (5)
S1—C20	1.727 (5)	C7—H7	0.9500
S1'—C17	1.695 (9)	C8—C9	1.408 (5)
S1'—C20'	1.713 (10)	C8—H8	0.9500
N1—C15	1.150 (5)	C9—C10	1.486 (5)
N2—C12	1.351 (5)	C10—C11	1.400 (5)
N2—H1	0.882 (10)	C11—C12	1.422 (5)
N2—H2	0.880 (10)	C11—C15	1.462 (5)
N3—C16	1.146 (5)	C12—C13	1.407 (5)
C1—C14	1.369 (5)	C13—C16	1.437 (5)
C1—C10	1.410 (5)	C13—C14	1.439 (5)
C1—C2	1.513 (5)	C14—C17	1.481 (5)
C2—C3	1.534 (5)	C17—C18	1.329 (6)
C2—H2A	0.9900	C17—C18'	1.367 (9)
C2—H2B	0.9900	C18—C19	1.445 (6)
C3—C4	1.531 (6)	C18—H18	0.9500
C3—H3A	0.9900	C18'—C19'	1.427 (9)
C3—H3B	0.9900	C18'—H18'	0.9500
C4—C5	1.364 (6)	C19—C20	1.354 (6)
C4—C9	1.408 (5)	C19—H19	0.9500
C5—C6	1.390 (6)	C19'—C20'	1.360 (9)
C5—H5	0.9500	C19'—H19'	0.9500
C6—C7	1.367 (6)	C20—H20	0.9500
C6—H6	0.9500	C20'—H20'	0.9500
			
C17—S1—C20	92.0 (2)	C1—C10—C9	118.4 (3)
C17—S1'—C20'	93.0 (5)	C10—C11—C12	121.9 (3)
C12—N2—H1	121 (2)	C10—C11—C15	124.5 (3)
C12—N2—H2	126 (3)	C12—C11—C15	113.5 (3)
H1—N2—H2	113 (4)	N2—C12—C13	121.8 (3)
C14—C1—C10	120.8 (3)	N2—C12—C11	121.2 (3)
C14—C1—C2	121.6 (3)	C13—C12—C11	117.0 (3)
C10—C1—C2	117.7 (3)	C12—C13—C16	117.9 (3)
C1—C2—C3	109.1 (3)	C12—C13—C14	121.2 (3)
C1—C2—H2A	109.9	C16—C13—C14	120.9 (3)
C3—C2—H2A	109.9	C1—C14—C13	119.6 (3)
C1—C2—H2B	109.9	C1—C14—C17	127.0 (3)
C3—C2—H2B	109.9	C13—C14—C17	113.3 (3)
H2A—C2—H2B	108.3	N1—C15—C11	172.9 (4)
C4—C3—C2	109.5 (3)	N3—C16—C13	176.6 (4)
C4—C3—H3A	109.8	C18—C17—C14	126.9 (4)
C2—C3—H3A	109.8	C18'—C17—C14	115.1 (11)
C4—C3—H3B	109.8	C18'—C17—S1'	111.3 (7)
C2—C3—H3B	109.8	C14—C17—S1'	133.6 (8)
H3A—C3—H3B	108.2	C18—C17—S1	111.5 (3)
C5—C4—C9	120.4 (4)	C14—C17—S1	121.6 (3)
C5—C4—C3	121.6 (4)	C17—C18—C19	113.4 (4)
C9—C4—C3	117.9 (4)	C17—C18—H18	123.3
C4—C5—C6	120.5 (4)	C19—C18—H18	123.3
C4—C5—H5	119.7	C17—C18'—C19'	112.4 (6)
C6—C5—H5	119.7	C17—C18'—H18'	123.8
C7—C6—C5	120.2 (4)	C19'—C18'—H18'	123.8
C7—C6—H6	119.9	C20—C19—C18	112.1 (4)
C5—C6—H6	119.9	C20—C19—H19	124.0
C6—C7—C8	120.5 (4)	C18—C19—H19	124.0
C6—C7—H7	119.8	C20'—C19'—C18'	112.7 (7)
C8—C7—H7	119.8	C20'—C19'—H19'	123.7
C7—C8—C9	119.6 (4)	C18'—C19'—H19'	123.7
C7—C8—H8	120.2	C19—C20—S1	111.1 (3)
C9—C8—H8	120.2	C19—C20—H20	124.5
C8—C9—C4	118.6 (4)	S1—C20—H20	124.5
C8—C9—C10	122.1 (3)	C19'—C20'—S1'	110.8 (8)
C4—C9—C10	119.3 (3)	C19'—C20'—H20'	124.6
C11—C10—C1	119.4 (3)	S1'—C20'—H20'	124.6
C11—C10—C9	122.2 (3)		
			
C14—C1—C2—C3	−135.7 (4)	C2—C1—C14—C13	−178.3 (3)
C10—C1—C2—C3	43.4 (5)	C10—C1—C14—C17	−174.1 (3)
C1—C2—C3—C4	−58.1 (4)	C2—C1—C14—C17	5.0 (6)
C2—C3—C4—C5	−142.1 (4)	C12—C13—C14—C1	−2.2 (5)
C2—C3—C4—C9	34.1 (5)	C16—C13—C14—C1	−179.3 (3)
C9—C4—C5—C6	−0.3 (6)	C12—C13—C14—C17	174.9 (3)
C3—C4—C5—C6	175.8 (4)	C16—C13—C14—C17	−2.2 (5)
C4—C5—C6—C7	−2.3 (6)	C1—C14—C17—C18	−118.5 (4)
C5—C6—C7—C8	1.5 (6)	C13—C14—C17—C18	64.6 (3)
C6—C7—C8—C9	1.8 (6)	C1—C14—C17—C18'	62.6 (12)
C7—C8—C9—C4	−4.3 (5)	C13—C14—C17—C18'	−114.3 (11)
C7—C8—C9—C10	175.3 (3)	C1—C14—C17—S1'	−117.4 (11)
C5—C4—C9—C8	3.6 (6)	C13—C14—C17—S1'	65.7 (11)
C3—C4—C9—C8	−172.7 (4)	C1—C14—C17—S1	61.6 (4)
C5—C4—C9—C10	−176.1 (4)	C13—C14—C17—S1	−115.3 (3)
C3—C4—C9—C10	7.6 (5)	C20'—S1'—C17—C18	−173 (8)
C14—C1—C10—C11	−0.1 (5)	C20'—S1'—C17—C18'	0.1 (3)
C2—C1—C10—C11	−179.2 (3)	C20'—S1'—C17—C14	−179.92 (19)
C14—C1—C10—C9	177.3 (3)	C20'—S1'—C17—S1	1.0 (10)
C2—C1—C10—C9	−1.8 (5)	C20—S1—C17—C18	−0.9 (2)
C8—C9—C10—C11	−28.0 (6)	C20—S1—C17—C18'	171 (9)
C4—C9—C10—C11	151.7 (4)	C20—S1—C17—C14	178.99 (15)
C8—C9—C10—C1	154.7 (4)	C20—S1—C17—S1'	−1.8 (8)
C4—C9—C10—C1	−25.6 (5)	C18'—C17—C18—C19	0.2 (14)
C1—C10—C11—C12	−3.0 (5)	C14—C17—C18—C19	−178.7 (2)
C9—C10—C11—C12	179.8 (3)	S1'—C17—C18—C19	8 (7)
C1—C10—C11—C15	172.9 (3)	S1—C17—C18—C19	1.2 (3)
C9—C10—C11—C15	−4.3 (6)	C18—C17—C18'—C19'	1.0 (12)
C10—C11—C12—N2	−178.5 (3)	C14—C17—C18'—C19'	180.0 (3)
C15—C11—C12—N2	5.2 (5)	S1'—C17—C18'—C19'	0.0 (3)
C10—C11—C12—C13	3.3 (5)	S1—C17—C18'—C19'	−8 (9)
C15—C11—C12—C13	−173.0 (3)	C17—C18—C19—C20	−1.0 (4)
N2—C12—C13—C16	−1.7 (5)	C17—C18'—C19'—C20'	−0.1 (5)
C11—C12—C13—C16	176.5 (3)	C18—C19—C20—S1	0.2 (4)
N2—C12—C13—C14	−178.9 (3)	C17—S1—C20—C19	0.4 (3)
C11—C12—C13—C14	−0.7 (5)	C18'—C19'—C20'—S1'	0.1 (6)
C10—C1—C14—C13	2.6 (6)	C17—S1'—C20'—C19'	−0.1 (5)

### Hydrogen-bond geometry (Å, º)

Cg1 is the centroid of the C4–C9 ring.

**Table:**

*D*—H···*A*	*D*—H	H···*A*	*D*···*A*	*D*—H···*A*
N2—H1···N3^i^	0.88 (3)	2.18 (3)	3.016 (5)	160 (3)
C6—H6···*Cg*1^ii^	0.95	2.85	3.660 (5)	144

Symmetry codes: (i) −*x*+1, −*y*, −*z*+1; (ii) −*x*+1, *y*+1/2, −*z*+3/2.
